# Recent Advances in Insect Rearing Methodology to Promote Scientific Research and Mass Production

**DOI:** 10.3390/insects12110961

**Published:** 2021-10-22

**Authors:** Man P. Huynh, Kent S. Shelby, Thomas A. Coudron

**Affiliations:** 1 Division of Plant Science & Technology, University of Missouri, Columbia, MO 65211, USA; 2 Department of Plant Protection, Can Tho University, Can Tho 900000, Vietnam; 3 Biological Control of Insects Research Laboratory, USDA-Agricultural Research Service, Columbia, MO 65203, USA

The benefits obtained from our ability to produce insects have encompassed a wide array of applications, from the early stages of examining different species, to the present day of mass production for multiple purposes. Perhaps the most prominent application to date is insect management for production agriculture. Much of the considerable knowledge humans have of insect biology has been made possible by first bringing insects from the field into the laboratory, and maintaining sufficiently large colony sizes on bespoke, empirically developed diets. The ability to conduct experiments without the seasonal limitations that normally bound insect life history, to control abiotic and biotic treatments, is heavily dependent upon a nutritionally complete, inexpensive and easily produced or procured diet acceptable to the insects. Nominally, healthy insects produced in this way are more likely to respond accurately in various bioassays, to better perform the desired ecosystem services upon release, or to act as nutritional inputs for other animals. Ecosystem services provided by mass reared insects include pollination, release of predators or parasitoids for biological control and releases of sterile insects to suppress pest populations. More recently, the usage of mass-produced insects for nutrient sources such as for human consumption, animal feed, pet food, stock chemicals, or valorization of waste streams has the potential to surpass all previous applications. Of course, food sources are not the minimum requirement for successful mass rearing. Attention must be paid to innovate and optimize many other inputs including reduced labor, close observation of losses at each life stage, mating and oviposition, appropriately shaped containers, lighting, scheduling and sanitation.

The initial effort of the mass production of insects probably has its origin in scientific inquiry. The production of healthy, vigorous experimental subjects for basic and applied research purposes was an early impetus for the development of insectary diets and rearing procedures. That desire to study insects remains and has been greatly assisted by the ability to produce the specimens under natural and controlled conditions. Typical of research efforts is the progression of developing, refining and adapting supportive procedures that enable advancements. Insect rearing is a prime example.

After collection, the need to rear becomes the dominant challenge in all efforts to produce insects and that requires an understanding of environmental and nutritional needs. Along with an increased knowledge of insects came technological advances that resulted in improved methodologies and commercially available supplies. Courses, symposia and conferences in insect rearing were held and as the knowledge-based methodologies and supportive materials expanded, so did the applications of rearing insects. An industry of insect production and sales emerged and continues today, as does the need for quality assurance associated with rearing, research and production.

Numerous insects have been reared and many continue to be reared today. Applications include discovery of insect pheromones, repellents and new compounds with insecticidal properties. Some are used for monitoring purposes, including detection of insect resistance. At the forefront of mass rearing was the emerging field of the sterile insect technique (SIT) for area-wide management of pest insects. Targeted insects included the screwworm fly, several tephritid fruit flies, the gypsy moth, pink bollworm, boll weevil, codling moth and several mosquito species. Many beneficial insects have been, and continue to be, reared for release as biological control measures against pest insects. The most notable of these may be *Trichogramma* species. The glasshouse industry has become heavily dependent on insectary reared insects for pest control. Today, we have an emerging field of insect production as biofactories, both for food and specialty substance sources. These new fields have tremendous potential.

Most agricultural applications of insect rearing require large-scale production. One of the major advancements that has enabled mass production of insects has been the substitution of alternative food sources, and in particular, artificial diets. Artificial diets allow for the mass production of insects in simpler, highly controlled, cost-effective and more convenient ways compared to rearing on native diets such as plants or natural preys. Ideal artificial diets can ultimately serve as suitable substitutes for natural foods and support the production of insects physically and behaviorally, similar to natural diets. Often, the use of artificial diets results in a more uniform and consistent production of high-quality insects for research and field applications. Consequently, the investigation of substitutes for natural food has become a common practice.

The application of new technologies is on the horizon for further refinements in insect production, such as genomics, genetic selection and engineering. Newer multi-omics technologies such as transcriptomics, nutrigenomics and nutrimetabolomics, with increased knowledge of insect microbiome contributions and statistical optimization modeling, have already enabled significant advances in diet formulation. These advances have resulted in a better understanding of the effects of the food stream ingredients on physiological and biochemical functions. Undoubtedly, this will result in improved diet formulations, higher quality control and healthier reared insects with better performance for their targeted applications.

The black soldier fly (*Hermetia illucens* L.) has a global research interest and a growing industrial application since it provides a viable option for countering the environment detriments caused by food waste and a sustainable protein source to feed the growing global population. Hopkins et al. [1] conducted a systematic literature review investigating the impacts of various foodstuffs and substrates for food waste rearing on the protein and amino acid composition of the black soldier fly larvae, finding that plant rearing substrates provide a lower protein content of the total larval mass compared to animal rearing substrates. Pliantiangtam et al. [2] investigated the growth performance, waste reduction efficiency and nutritional composition of the black soldier fly larvae reared on two plant materials (coconut endosperm and soybean curd residue), reporting a similar result of the use of plant rearing sources that yielded lower protein larvae compared with that of animal rearing materials. Furthermore, Lu et al. [3] compared the effects of nine nitrogen sources (i.e., NH_4_Cl, NaNO_3_, urea, uric acid, Gly, L-Glu, L-Glu:L-Asp (1:1, *w*/*w*), soybean flour, and fish meal) during food waste larval treatment and characterized the C/N effects on the larval development and bioconversion process. The authors found that organic nitrogen was more suitable than NH_4_Cl and NaNO_3_ as the nitrogen amendment, and an inclusion of small amounts of urea (C/N of 18:1–14:1 and 18:1–16:1) improved the waste reduction performance, and larval protein and lipid bioconversion process, respectively.

The yellow mealworm (*Tenebrio molitor* L.) is another insect species that has been considered as an alternative to fishmeal in animal feed formulations. By utilizing a nutrient self-selection approach, Morales-Ramos et al. [4] demonstrated that the optimum ratio of macro-nutrient intake of this species was 0.06:0.23:0.71 for lipid:protein:carbohydrate. Carbohydrate had positive impacts on food assimilation, food conversion and biomass gain, and several plant materials including cabbage, potato, wheat bran, rice bran (whole and defatted), corn dry distillers’ grain, spent brewery dry grain, canola meal and sunflower meal were suitable macro-nutrient components in *T. molitor* diets.

Nikolouli et al. [5] evaluated inactive *Enterobacter* sp. AA26 as a protein source to potentially replace the inactive brewer’s yeast, a protein ingredient in the larval diet of the spotted-wing drosophila fly (*Drosophila suzukii* (Matsumura)). This insect species is one of the most damaging insect pests of soft skinned fruits in North America and Europe and is a detrimental invasive pest in South America and Africa. The authors found that *Enterobacter* sp. AA26 provided inadequate nutrition in the larval diet compared with the inactive brewer’s yeast. The replacement of *Enterobacter* sp. AA26 with the inactive brewer’s yeast resulted in decreases in pupal weight, survival, fecundity and adult recovery.

Improvements in the methodology to store beneficial insects at low temperatures will facilitate biological control programs primarily relying on the mass-release of high-quality bioagents to suppress agricultural pests. Lin et al. [6] characterized impacts of temperatures (4, 7, 10 and 13 °C) and storage durations (10, 15, 20 and 25 days) on the developmental parameters of different pupal age of *Psyttalia incisi* (Silvestri), a dominant parasitoid against *Bactrocera dorsalis* (Hendel) in fruit-producing regions of southern China. The authors reported that the emergence rate of *P. incisi* was significantly affected by storage temperature, storage duration and pupal age interval and their interactions. They further determined the optimum cold storage conditions at a temperature of 13 °C for 10 or 15 d for late-age pupae of this parasitoid. Separately, Lü et al. [7] demonstrated that a temperature of 13 °C was the cold tolerance threshold temperature and the prepupal stage was a critical developmental period for in vitro rearing of another parasitoid *Trichogramma dendrolimi* Matsumura, an important biological control agent of biological control programs.

Insect predators are also important components of biological control programs. Zou et al. [8] investigated the effects of prey species (*Drosophila melanogaster* Meigen or *Bradysia impatiens* (Johannsem)) and prey densities (6–48 preys per predator) on the performance of adult *Coenosia attenuate* Stein, a predator species native to Southern Europe effectively suppressing a wide range of agricultural pests. Their results revealed that *B. impatiens* was the better prey compared with *D. melanogaster*, and the optimal prey density for *C. attenuate* rearing was 12 to 24 adults of *B. impatiens* per predator per day.

Rearing honeybee (*Apis mellifera*) larvae in vitro is an important method for studying bee larvae diseases or the toxicity of pesticides on bees. Kim et al. [9] evaluated the emergence and deformation rates of honeybee (*Apis mellifera ligustica* Spinola) larvae reared in horizontal and vertical positions, finding that a vertical rearing method was the better approach. Compared to the horizontal rearing plates, the vertical rearing plates resulted in a twofold decrease in adult deformation rates and larger adults (11.6 mm vs. 10.8 mm).

Heat-sterilized diets are the key components of high-throughput systems for mass production of insects. Huynh et al. [10] investigated the influence of thermal exposure and lengths of thermal exposure on the quality of a commercialized diet for the western corn rootworm (*Diabrotica virgifera virgifera* LeConte), the most serious pest of maize in the United States and some parts of Europe, to further the goal of developing a diet free of antibiotics and heat-sterilized for this important pest. By using geometric and mathematical approaches, the authors demonstrated non-linear effects of thermal exposure on the performance of diet, whereas no impacts were observed on the exposure intervals evaluated. These findings will guide the continued development of sterilized rootworm diets, facilitating mass production and providing insights into the design of diets for other insects.

This unique topic has been captured in 10 articles that bring together experimental and review papers focusing on different rearing technology approaches to many facets of insect rearing for various purposes. There is every reason to believe that the rapid improvements in insect nutrition and rearing seen over the past decade will be dwarfed by the accomplishments yet to come in the next decade.
